# Establishing Minimal Conditions Sufficient for the Development of Titan-like Cells in *Cryptococcus neoformans*/*gattii* Species Complex

**DOI:** 10.3390/pathogens11070768

**Published:** 2022-07-05

**Authors:** Mariusz Dyląg, Rodney J. Colón-Reyes, Yaliz Loperena-Álvarez, Lukasz Kozubowski

**Affiliations:** 1 Department of Genetics and Biochemistry, Clemson University, Clemson, SC 29634, USA; rcolonr@g.clemson.edu; 2 Department of Mycology and Genetics, Faculty of Biological Sciences, University of Wroclaw, 51-148 Wroclaw, Poland; 3 Department of Science, Pontifical Catholic University of Puerto Rico-Mayagüez Campus, Mayagüez PR 00681, Puerto Rico; yaliz_loperena@pucpr.edu

**Keywords:** Titan cells, yeast-like fungi, virulence factors, pathogenesis, cryptococcosis

## Abstract

Opportunistic pathogens of the anamorphic genus *Cryptococcus* are unique considering their virulence factors that in the context of pathogenesis allowed them to achieve evolutionary success. Morphological transformation into giant (Titan) cells is one of the factors contributing to cryptococcosis. Recently established in vitro protocols demonstrate that 5 or 10% fetal bovine serum (FBS) combined with 5% CO_2_, 37 °C, and sufficiently low cell density, triggers cellular enlargement (Serum protocols). However, the FBS components that promote this morphological transition remain incompletely characterized. In search of minimal conditions necessary for stimulating the formation of Titan cells, we performed a study where we eliminated serum from the protocol (Serum-free protocol) and instead systematically adjusted the amount of glucose, source of nitrogen (ammonium sulfate), and the pH. We found that exposing cells to PBS with adjusted pH to 7.3, and supplemented with 0.05% glucose, 0.025% ammonium sulfate, 0.004% K_2_HPO_4_, 0.0035% MgSO_4_, in the presence of 5% CO_2_ at 37 °C triggers Titan-like cell formation to the same degree as the previously established protocol that utilized 10% FBS as the sole nutrient source. Titan-like cells obtained according to this Serum-free protocol were characterized by cell body size over ten microns, a single enlarged vacuole, thick cell wall, extensive polysaccharide capsule, and changes in the level of cell ploidy, all currently known hallmarks of Titan cells found in vivo. Strikingly, we found that in both, Serum and Serum-free protocols, an optimal pH for Titan-like cell development is ~7.3 whereas relatively acidic pH (5.5) prevents this morphological transition and promotes robust proliferation, while alkaline pH (~8.0) has a profound growth inhibitory effect. Our study demonstrates a critical role of pH response to the formation of Titan cells and indicates that conditions that allow restricted proliferation in the presence of 5% CO_2_ are sufficient for this morphological transition to form enlarged cells in *Cryptococcus neoformans* and *Cryptococcus* *gattii* species complex.

## 1. Introduction

Species belonging to the *Cryptococcus neoformans* and *Cryptococcus gattii* complex are one of the most important etiological agents of invasive fungal infections (IFIs) accompanied by high mortality [1,2]. Almost one million cases of cryptococcal infections of the central nervous system (CNS) occur every year resulting in more than 600,000 deaths including 15–17% of AIDS-related deaths worldwide [3,4]. While members of the *C. neoformans* species complex can cause mostly opportunistic infections in immunocompromised individuals, representatives of the *C. gattii* species complex are able to also infect immunocompetent persons [5,6,7,8].

Members of the *C. neoformans*/*C. gattii* species complex are able to undergo morphological transition and form enlarged cells termed Titan cells, which have been shown to enhance dissemination and persistence in the host [9,10]. When grown in vitro in rich media, the diameter of the cells of *C. neoformans*/*C. gattii* species complex is within the range from 4 to 6 µm. In contrast, Titan cells are larger than 10 µm, possess a thicker cell wall due to the additional layer of chitin, single centrally located large vacuole, and single nucleus characterized by increased ploidy [11,12,13]. Titan cells are also more resistant to phagocytosis [9,11,14,15] and aneuploid daughter cells of *C. neoformans* Titan cells show increased resistance to environmental stress, including exposure to antifungal drugs [10]. The role of Titan cells in pathogenesis is unclear, as some studies show Titan cell production impacts virulence in animal models, but this impact in human studies is less clear because strains that do not form Titan-like cells in vitro have been isolated from humans [16,17,18,19]. Nonetheless, the recent study suggests that the formation of Titan cells is unique to pathogenic *Cryptococcus* species, as a similar morphological transition has not been detected in vitro in several species that do not belong to the *C. neoformans* and *C. gattii* species complexes [20].

External physicochemical factors that stimulate the formation of Titan-like cells and genes that are crucial for the development of Titans are not fully established [16,17,18]. Pathways shown to be involved in this process include the cyclic adenosine monophosphate (cAMP) signaling (dependent on the transcription factor Rim101) and the mating pathway [13,21,22]. Critical for our understanding of the mechanisms of triggering the formation of Titan cells are recently established protocols that allow obtaining Titan-like cells in vitro [16,17,18]. These studies indicated that Titan cell formation can be stimulated or is dependent on different external signals like CO_2_, hypoxia, and incubation in the presence of fetal bovine serum (FBS) [16,17,18]. Among constituents of the FBS, phospholipids, and bacterial cell wall components were shown to stimulate cellular enlargement [16,18,23]. Consistently, Titan-like cells have been observed in *C. neoformans* grown in YNB (yeast nitrogen base medium) when co-cultured with bacteria [18]. However, Titan-like cells have been also obtained under conditions of continual four-day incubation of *C. neoformans* in YNB medium alone, although Titan-like cells obtained under these conditions reached sizes that were relatively smaller as compared to Titan cells obtained in vivo [11]. Furthermore, a recent study indicates that transition to Titan cells is stimulated by the G2 cell cycle arrest followed by a return to the cell cycle with reduced expression of the cyclin gene *CLN1* [24].

Based on these studies, we hypothesized that optimal for the formation of Titan-like cells in vitro are conditions that sufficiently restrict proliferation in the presence of 5% CO_2_. To validate this hypothesis, we systematically tested representative members of the *C. neoformans*/*C. gattii* species complex for their ability to undergo this unique morphological transformation when cells are brought from poor-nutrient media to fresh PBS-based media with decreasing levels of nutrients at various pH values in the presence of 5% CO_2_ at 37 °C. We found an optimal level of nutrients and the pH that allow the formation of Titan-like cells to the same extent as the recently established protocol that required the addition of the FBS. Our data indicate that minimal conditions sufficient to trigger the development of Titan-like cells in vitro include 5% CO_2_, 37 °C, pH of ~7.3, and the amount of glucose and source of nitrogen below a specific threshold.

## 2. Results

### 2.1. A Critical Role of the pH in the Formation of Titan Cells

**Figure 1:**
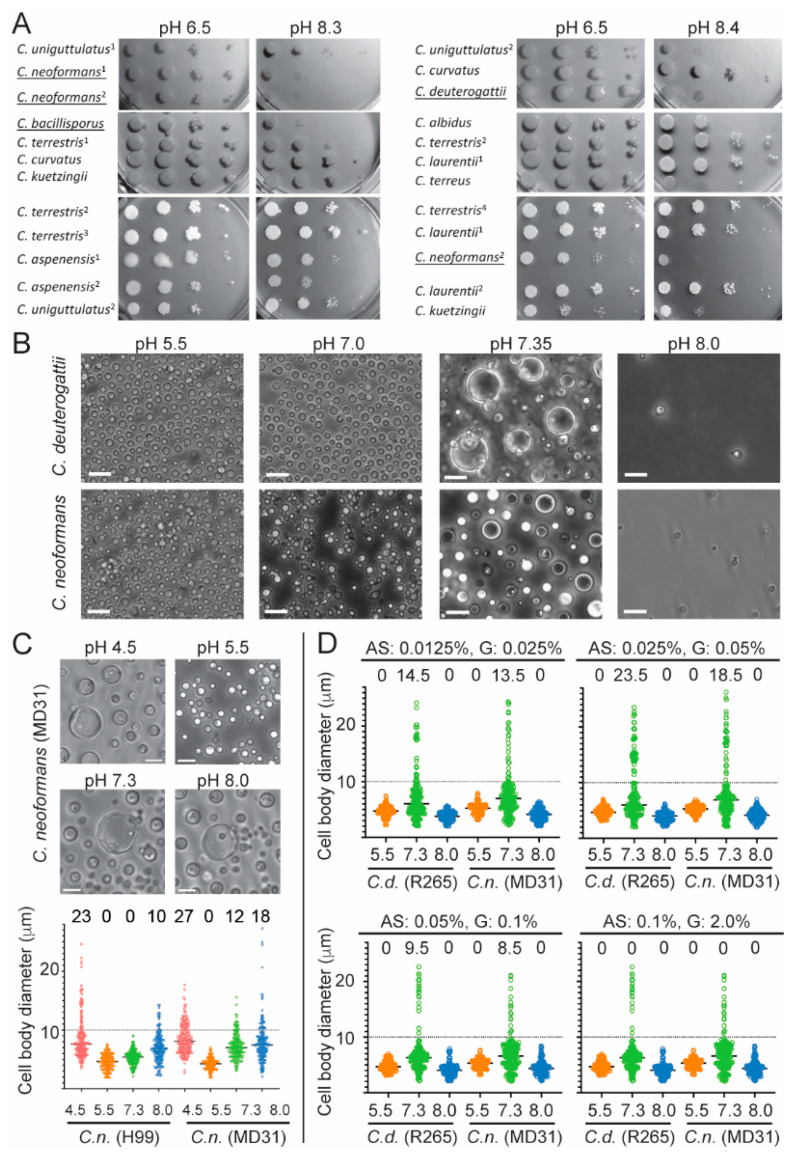
The effect of pH and nutrient level on the formation of Titan cells. (**A**) A spot test assay was utilized to test the sensitivity to alkaline pH of representative strains (alternative strains of the same species are indicated by numbers and are characterized in detail in Table 2) of the non-*neoformans* and non-*gattii* species as compared to four representative strains (underlined) of the *C. neoformans*/*C. gattii* species complex. Experiments based on spot test assay were performed in two independent repetitions. (**B**) The level of pH is critical for the formation of Titan-like cells in *C. neoformans* (H99) and *C. deuterogattii* (R265) in a modified “Serum” protocol based on a method developed by Dambuza I.M. et al. [18]. (**C**) The level of pH (4.5, 5.5, 7.3, and 8.0) influences the percentage of Titan cells obtained in a modified protocol based on the method established by Hommel B. et al. [17]. The formation of Titan-like cells was tested for two representatives of the *C. neoformans* (strains MD31 and H99), and at least 200 cells were measured for each sample. Differences in cell body diameter between the experimental groups were significant (one-way ANOVA, *p* = 0.001). (**D**) Optimal conditions for the development of Titan-like cells were established in a Serum-free protocol. A method developed by Dambuza I.M. et al. has been modified such that the fetal bovine serum (FBS) has been replaced by various levels of nutrients (ammonium sulfate: AS, glucose: G) and pH levels (5.5, 7.3, 8.0), as indicated (see text for more details). Results are shown for *C. deuterogattii* strain R265 and *C. neoformans* strain MD31. For each strain and condition, at least 200 cells were measured. Above the plot, for each sample, the percentage of Titan cells (cell body diameter above 10 microns) is indicated. For each of the four versions of the nutrient content, the median cell body diameter at pH = 7.3 was significantly different from the diameter of cells incubated at pH 5.5 or 8.0 (Kruskal-Wallis test, *p* = 0.001). Scale bars are 20 microns in B and 10 microns in C.

A recent study suggested that the members of the *C. neoformans*/*C. gattii* species complex, among the other basidiomycetous yeasts, are uniquely capable of forming Titan cells, but the basis for this presumed distinct characteristic remains unclear [20]. The Rim101 transcription factor, which is involved in the cellular response to changes in pH, has been implicated in the formation of Titan cells [25]. A recent study has demonstrated the influence of the pH on the formation of Titan-like cells in vitro based on a protocol that involved exposing stationary phase cells of *C. neoformans* to conditions with limited aeration [17]. To evaluate the potential correlation between response to pH and the ability to form giant cells, we tested 8 non-*neoformans*/non-*gattii* basidiomycetous species for their sensitivity towards pH. We noted that four strains (representing three species) of the *C. neoformans*/*C. gattii* species complex were relatively more sensitive to alkaline pH as compared to 11 strains (representing 6 species) of non-*neoformans*/non-*gattii Cryptococcus* species (Figure 1A). This observation that the species sensitive to high pH are the species that form Titan cells led us to test the impact of pH on in vitro Titan-like cell production. We modified the protocol developed by Dambuza et al. that induces Titan cell formation using fetal bovine serum (FBS) to include exposing cells to various pH levels [18]. We found that pH significantly influenced Titan-like cell formation after 48 h of incubation of two representative members of the *C. neoformans* and *C. gattii* species complexes (Figure 1B). Specifically, at pH 5.5, no Titan-like cells were formed and the cultures proliferated abundantly. At pH 7.0, proliferation was still relatively high, and no Titan-like cells were obtained. Strikingly, at pH~7.3, less proliferation was observed, and Titan-like cells were formed. Increasing the pH to ~8.0 resulted in drastic inhibition of growth and a lack of cellular enlargement (Figure 1B). Finally, we also investigated how initial inoculum size affects the pH of culture after 48 h incubation and the total number of cells under conditions based on the Dambuza et al. protocol [18]. After 48 h of incubation in case of the initial inoculum established on the level of 10^4^ cells/mL, we could find significantly greater differences between the pH values respectively for time T_0_ and T_48_, indicating faster and more pronounced acidification of the medium compared to the results obtained for the initial inoculum equal to 10^3^ cells/mL (Table 1). Among the four strains tested of the *C. neoformans*/*C. gattii* species complex, only the *C. bacillisporus* B4546 strain achieved a much lower density (defined as the number of cells per milliliter) and acidified the culture to a much lesser extent (10% FBS in PBS). This suggests that this strain is more sensitive to even slightly alkaline pH (~7.2) and proliferates particularly well at acidic pH. Moreover, strain B4546 under such conditions is able to form Titan cells with high efficiency, as has been demonstrated previously [20]. The size of the initial inoculum also influenced the efficiency of the Titan cell formation, namely the percentage of Titans was smaller when starting inoculum was equal to 10^4^ cells/mL as compared to 10^3^ cells/mL (Table 1).

An alternative in vitro protocol to obtain Titan-like cells has been established that does not include exposure to FBS and 5% CO_2_ [17]. In this protocol, established by Hommel B. et al., cellular enlargement occurs when stationary phase cells are exposed to conditions with restricted aeration at 30 °C [17]. Evaluating four levels of pH in this protocol (4.5, 5.5, 7.3, and 8.0) revealed that *C. neoformans* (H99 and MD31) did not form Titan-like cells at pH 5.5 and the highest percentage of Titan-like cells was obtained at extreme pH (4.5 and 8.0) (Figure 1C). At pH 7.3, the *C. neoformans* strain H99 did not form Titan-like cells, whereas the *C. neoformans* strain MD31 underwent this transition (Figure 1C). Interestingly, when using the protocol established by Hommel B. et al., no cellular enlargement was observed in the case of four representatives of the *C. gattii* species complex (*C. bacillisporus* strains WM161, B4546, NIH312 and *C. deuterogattii* strain R265) under the four levels of pH tested (data not shown). Thus, pH plays a critical role during the formation of Titan-like cells in vitro and the optimal pH level differs between conditions that include 10% FBS and 5% CO_2_ [18] and those that expose the cells to hypoxia-like conditions [17]. Furthermore, a relatively high level of sensitivity to alkaline pH may be contributing to the unique ability of the members of the *C. neoformans* and *C. gattii* species complexes to form Titan cells.

### 2.2. Establishing Optimal Conditions to Obtain Titan-like Cells In Vitro

**Figure 2:**
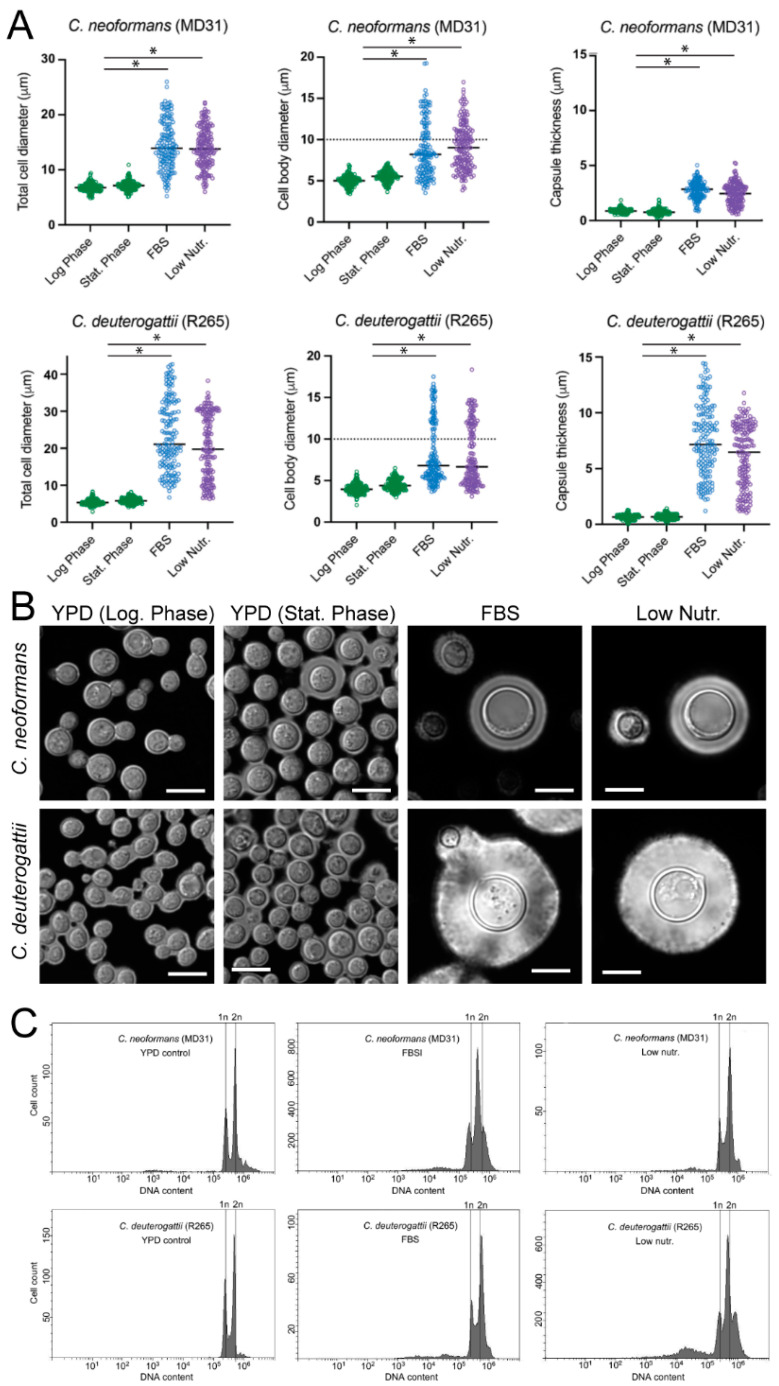
A direct comparison of the percentage of Titan cells obtained in an optimized Serum-free protocol (Low nutr., 0.025% ammonium sulfate, 0.05% glucose) as compared to the method developed by Dambuza I.M. et al. (FBS) [18]. (**A**) *C. neoformans* strain MD31 and *C. deuterogattii* strain R265 were utilized. As controls, cultures grown in a rich YPD medium at logarithmic (Log. Phase, shown in A and C) or stationary phase (Stat. phase, shown in A) of growth were evaluated. For each sample, the cell/capsule diameter for at least 150 cells was measured. Star indicates statistical significance (*p* = 0.001). (**B**) Cells evaluated in A were stained with India ink to visualize the capsule. (**C**) Nuclei of cells evaluated in A were stained with SYTOX^®^ Green and fluorescence flow cytometry was performed to examine ploidy. The Y-axis scale was adjusted in each panel to facilitate the comparison of peaks for each sample. The scale bar in B represents 10 microns.

**Figure 3:**
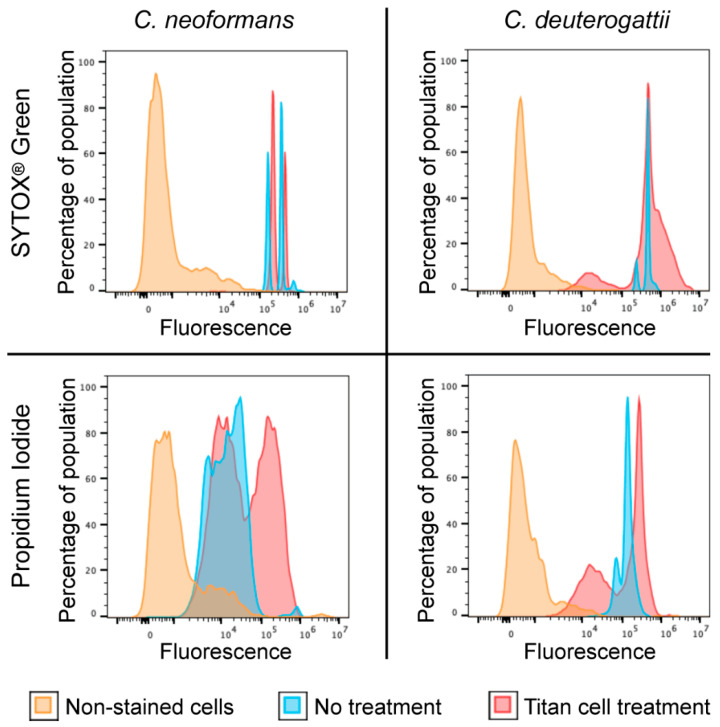
Analysis of the DNA content in cells grown in YNB medium (control, blue) or cells subject to Serum-free protocol (red). Cells were stained for DNA content with either SYTOX^®^ Green or propidium iodide. Yellow indicates additional control of cells that were not stained for the DNA content. The analysis was performed for *C. neoformans* MD31 and *C. deuterogattii* R265 strains. The percentage of the population was normalized to mode.

A common factor between the two established in vitro protocols that included the FBS [16,18] seems to be the transfer of cells to the culture media that limit robust proliferation [11,26]. It is commonly known that serum can induce capsule growth [26] but also cellular enlargement [16,18]. Moreover, serum is most effective in inducing enlargement under nutrient-limited media [26]. This presumed limitation of proliferation may be due to relatively low nutrient content of the FBS and/or some specific FBS components that exhibit an inhibitory effect on growth and/or due to suboptimal pH. We hypothesized that replacing FBS by defined nutrient composition at levels sufficiently low to restrict proliferation may trigger cellular enlargement. To test this possibility, we modified the protocol established by Dambuza I.M. et al. [18], by eliminating the FBS and instead supplementing phosphate-buffered saline (PBS, which was originally included in the protocol) with glucose and a nitrogen source (ammonium sulfate) at decreasing four concentrations (Serum-free conditions). We also established that it was necessary to supplement the medium with inorganic magnesium and phosphate (0.007% K_2_HPO_4_ and 0.008% MgSO_4_), otherwise, there was no proliferation observed (data not shown). Importantly, we tested three levels of pH for each of the nutrient levels. This “checkerboard” type of assay led to the identification of optimal conditions for the formation of Titan-like cells with respect to pH and nutrient content (Figure 1D). Specifically, we found that adjusting the PBS to a pH of ~7.3, and adding 0.05% glucose, 0.025% ammonium sulfate, 0.007% K_2_HPO_4_, and 0.008% MgSO_4_, in the presence of 5% CO_2_ at 37 °C results in conditions that trigger Titan-like cell formation to the highest levels (Figure 1D). These conditions were optimal for two strains, namely *C. neoformans* MD31 and *C. deuterogattii* R265 (Figure 1D). This result suggested that FBS is not a necessary component to trigger the formation of Titan-like cells at 37 °C in the presence of 5% CO_2_. However, it remained possible that FBS could have an effect that stimulates this morphological transition to a larger extent than the optimal conditions established here. To test this possibility, we evaluated two strains: *C. neoformans* strain MD31, and *C. deuterogattii* strain R265, for their ability to develop into Titan-like cells under Serum (a protocol according to Dambuza I.M. et al. [18]) and the optimal Serum-free conditions established here (Figure 2A,B). Strikingly, for both species, Titan-like cells were induced to a similar extent in both protocols (Figure 2A,B). Importantly, both protocols led to Titan-like cells characterized by cell body diameter above 10 µm, enlarged central vacuole, thick cell wall, enlarged capsule (Figure 2A,B), and changes in the level of cell ploidy (Figure 2C), all hallmark features of Titan cells found in vivo. For cells subject to both protocols for 48 h, DNA content was estimated based on staining with the SYTOX^®^ Green stain (Figure 2C). In addition, cells were subject to Serum-free protocol for 5 days and DNA content was estimated based on SYTOX^®^ Green and propidium iodide staining for comparison (Figure 3). This alternative staining method further confirmed an increase in DNA content when cells were subject to the Serum-free protocol (Figure 3). Interestingly, an average capsule thickness was higher in *C. deuterogattii* R265 cells as compared to *C. neoformans* MD31 when the cells were treated by either of the two protocols, which has contributed to an overall larger total cell diameter in R265 as compared to strain MD31 (Figure 2A,B). In addition, when the Serum-free protocol was further modified by changing the initial incubation from YNB (poor nutrient) medium to YPD (nutrient-rich medium), no Titans were formed, a result similar to that obtained in the previously established Serum protocol [18] (data not shown).

## 3. Discussion

Collectively, our results clearly indicate that the formation of Titan-like cells can be triggered in vitro when cultures are brought from poor nutrient media (YNB) to conditions that restrict robust proliferation. For instance, this can occur when cells are exposed to sufficiently low nutrients at a pH that is not optimal for proliferation under 5% CO_2_ at 37 °C, or when cells are exposed to hypoxia-like conditions. While previous studies have provided compelling evidence that specific component/s in the FBS can stimulate the development of Titan-like cells, our data indicate that FBS is not essential for this morphological transition if cells are brought from poor nutrient media at sufficiently low density to fresh media that contain the number of nutrients that is sufficiently low to limit proliferation, the pH is ~7.3, and the incubation is under 5% CO_2_ at 37 °C.

Two striking aspects are commonly observed when the formation of Titan-like cells is triggered in vitro. First, typically, a relatively small fraction of the cell population undergoes this morphological transition. Zaragoza O. et al. provided compelling evidence that mostly old cells in the population are those that undergo cellular enlargement, which may explain a small fraction of Titan-like cells often reported in other studies [11]. Second, it has been reported that the percentage of Titan-like cells formed in vitro varies significantly depending on a specific strain. For instance, over 80% of the population of *C. bacillisporus* strain B4546 exhibited this morphological transition in vitro according to Serum protocol [20]. On the other hand, the same strain failed to exhibit cellular enlargement under modified Serum-free conditions presented here (data not shown). These findings suggest that to form Titan cells, the population must be exposed to conditions that restrict robust proliferation, which would depend on incubation conditions and the type of cells/strains. Considering recent findings pointing to a critical role of cyclin Cln1 in the formation of Titan cells [24], it is possible that a “potency” to re-enter the cell cycle, dependent on the levels of the cyclins, determines whether a cell enters a route to become a Titan. Accordingly, old cells in the population would have a low potential to proliferate upon cell cycle re-entry. In addition, cell physiological factors that determine the formation of Titan cells are likely multifactorial. For instance, a formation of a large vacuole may play an important role in this process. In support of this possibility, we observe that the *C. bacillisporus* strain B4546 exhibits a large proportion of cells with enlarged central vacuoles already during the initial incubation in the YNB medium, a stage when no enlarged cells (over 10 µm size) are yet formed, which contrasts with other strains tested that show no large vacuoles at this initial stage of the protocol (data not shown). The potential role of the formation of large vacuoles is consistent with the influence of the pH. Furthermore, it has been demonstrated that formation of Titan-like cells in vitro in the presence of the FBS requires sufficiently low cell density [16]. Similarly, both for Serum-protocol [18] as well for the Serum-free protocol we observed that each of the two mentioned requires a cell density of ~1000 cells/mL to effectively lead to cellular enlargement (Table 1). It has been suggested that quorum sensing is important for the formation of Titans [17]. However, a non-exclusive alternative explanation of this relationship could be that at higher densities, cells can acidify the medium to the degree sufficient to stimulate proliferation and prevent cellular enlargement. This conclusion is supported by the results obtained in the experiment performed according to the protocol established by Dambuza et al. [18]. Our data indicate that a higher density of initial inoculum, i.e., 10^4^ cells/mL resulted in a lower pH of the culture and a lower percentage of Titans after 48 h as compared to initial inoculum size equal to 10^3^ cells/mL (Table 1).

Data from this study suggest that the members of the *C. gattii* species complex do not form Titan-like cells under conditions previously established by Hommel B. et al. [17]. This result suggests that in contrast to older cells in the population of the *C. neoformans*, cells of the members of the *C. gattii* species complex proliferate upon re-entry of the cell cycle at a higher rate when exposed to hypoxia-like conditions, which may prevent those cells from entering a path to develop into Titan-like cells. This hypothesis is also based on the results obtained by Altamirano et al. [24], however, a study that compares the ability to re-enter the cell cycle between those species will be needed to test this possibility.

The uniqueness of members of the *C. neoformans*/*C. gattii* species complex to form Titan-like cells under established conditions described previously [20] emphasizes the evolutionary success of these fungi to develop as human pathogens. Our results suggest that what accounts for the ability to form Titan cells is increased sensitivity to alkaline pH. Given that the host internal body’s physiological pH is ~7.3, and the lung environment is deprived of nutrients available for stationary phase fungal cells that are inhaled from the environment or released from immune suppression, such an environment is predicted to trigger the formation of Titan cells. Further studies should establish common cellular features that promote Titan cell formation and will further explain the basis for strain and species-based differences in this peculiar morphological transition.

## 4. Materials and Methods

### 4.1. Tested Strains and Media

All the tested strains utilized in this study are listed in Table 2. Strains were cultured on YPD (Yeast extract Peptone Dextrose) semi-solid medium, followed by liquid YPD medium: 1% yeast extract, 2% bacto-peptone, and in case of semi-solid media 2% bacto-agar (BD Difco, Sparks, MD, USA, cat. no. REF212720, REF211820, and REF212720) and filter-sterilized 2% glucose (VWR International LLC, West Chester, PA, USA, cat. no. BDH0230). For the Titan induction experiment, cells were routinely grown overnight in 5 mL YNB (Yeast Nitrogen Base) liquid medium: 0.67% Yeast Nitrogen Base with amino acids, pH 5.5 (Sigma cat. no. Y1250, St. Louis, MO, USA), 2% glucose with horizontal shaking at 220 rpm and with incubation at 30 °C (Thermo Scientific, MAXQ4450, Waltham, MA, USA).

### 4.2. In Vitro Titan Cells Induction

Titan cells were obtained in vitro according to recently described protocols [17,18]. To follow the protocol established by Dambuza et al. [18], experiments were performed in sterile, biologically active 10% Fetal Bovine serum (FBS, Sigma, cat. no. F6178) diluted by 1× concentrated PBS (Dulbecco‘s Phosphate-Buffered Saline w/o Ca^2+^ and Mg^2+^, cat. no. REF21-031-CV, Corning cellgro^®^, Manassas, VA, USA), at final pH established on the level 7.4. Original concentrated FBS was normally stored in 2.5 mL aliquots at −20 °C to avoid repeated freeze-thaw procedure. Cryptococcal cells were incubated in static conditions for 48 h at 37 °C and under 5% CO_2_ atmosphere (New Brunswick an Eppendorf company, Galaxy 170S, Ayrshire, Scotland). To follow the protocol established by Hommel et al. [17], cells at final concentration 10^7^ cells/mL were initially incubated in 10 mL of the YPD liquid medium in flat bottle flasks at 30 °C with horizontal shaking at 150 rpm (Thermo Scientific, MAXQ4450) for 22 h. Next, cultures were centrifuged (5 min, 3000× *g*, room temperature) and the cells were washed twice using sterile MM medium: 15 mM D-glucose, 10 mM MgSO_4_, 29.4 mM KH_2_PO_4_, 13 mM Glycine and 3.0 µM Thiamine (Sigma cat. no. G8270, M7506, P5655, G7126 and T4625, respectively). In the final stage, cells were resuspended in 1 mL of MM medium in 1.5 mL Eppendorf tubes to obtain density equal to 10^6^ cells/mL (calculations performed based on cell counting using hemocytometer). Cells were incubated simultaneously in MM medium with pH established on the level 4.5, 5.5, 7.3 and 8.0. Cell suspensions prepared in this way were incubated for up to 5 days at 30 °C with horizontal shaking at 800 rpm (Eppendorf ThermoMixer, Eppendorf, Enfield, CT, USA). For the optimized Serum-free protocol, experiments were performed according to the protocol by Dambuza et al. [18] except that 10% FBS was replaced by low nutrient medium (0.025% ammonium sulfate, 0.05% glucose, 0.007% K_2_HPO_4_ and 0.008% MgSO_4_) and pH was adjusted to 7.3. To obtain these optimal conditions, initially the influence of different pH and various concentrations of components of the mentioned low nutrient medium (glucose and ammonium sulfate) were evaluated in a checkerboard-like assay in sterile 12-well microtiter plates (VWR^®^, Radnor, PA, USA), following the standard protocol [27].

### 4.3. Evaluation of Cell Viability

Each time viability of cells was routinely evaluated qualitatively by examination of micro-wells of the titration plate directly under the light microscope and quantitatively by plating on YPD medium. For this purpose, after mixing the contents of each micro-well of the titration plate, 100 µL volume was collected and either initially diluted (if necessary) using sterile 1× concentrated PBS or directly spread on YPD semi-solid medium. Plates were routinely incubated for 48 h at 30 °C in the incubator w/o CO_2_ (Thermo Scientific, Heratherm Incubator IMH180, Langenselbold, Germany). After this time, plates were photographed and colony counting was performed both to assess viability of cells as well as to determine total number of cells after the terminal point of the experiment.

### 4.4. Negative Staining with India Ink

Capsule visualization was performed based on negative acidic staining method with India ink. This technique permits visualization of the transparent and unstainable capsule in case of *Cryptococcus* spp. [28]. For this purpose microscopic preparations were performed as follows, 5 µL of India ink solution and 5 µL of yeast liquid culture was mixed on the surface of microscopic slide and then covered with a cover glass. All the microscopic preparations were examined using immersion oil and 100× objective under light microscopy using Leica DMi8 inverted microscope (Leica Microsystems Inc., Buffalo Grove, IL, USA).

### 4.5. Flow Cytometry to Determine Ploidy of Cells

Cell suspension obtained from the experiment to induce Titan cells was transferred into a 2 mL microfuge tube at a final concentration of cells equal to ~10^5^ cells/mL. Cells were harvested by centrifugation (30 s, 13,000× *g* at ~25 °C). Supernatants were removed and cells were resuspended in 1 mL 70% ethyl alcohol (EtOH). Cells were then incubated at ~25 °C for 1 h and transferred to 4 °C overnight. The next day, cells were centrifuged for 1 min and supernatant was removed. Cell pellets were briefly washed with 1 mL citrate solution (0.017% citric acid, 1.44% sodium citrate, Sigma cat no. 251275 and PHR1416, respectively), pH 7.0, and resuspended in 1 mL of freshly-prepared citrate solution with RNase A (0.25 mg/mL, Sigma cat. no. R4875) and SYTOX^®^ Green (500 nM, Invitrogen cat. no. S7020). Samples were covered with aluminum foil and left at 4 °C overnight. The next day immediately before analysis, cells were sonicated at an amplitude of 20% for ~10 s to avoid clumping and analyzed via fluorescence flow cytometry. For ploidy analysis, SYTOX^®^ Green fluorescence data were collected from 10,000 cells on a Beckman Coulter Cytoflex flow cytometer using the 488 nm blue laser with emission in the 530/30 BP filter. For the DNA content analysis based on propidium iodide (PI) staining, cells were fixed with EtOH for 24 h followed by staining using PI solution (5 µg/mL) with constant shaking at room temperature for 40 min. PI was removed by 1 min centrifugation at 5000 rpm and the cells were left overnight at 4 °C in PBS.

### 4.6. Microscopy and Imaging

All the microscopic observations were performed using inverted microscope (Leica DMi8, Leica Microsystems Inc., Buffalo Grove, IL, USA) using a 20×, 40× or 100× objectives and built-in camera for photographic documentation. Photographs were analyzed using Leica Application Suite X (LAS X) software (Leica Microsystems Inc., Buffalo Grove, IL, USA). For the assessment of cell body diameter, at least 150 cells for each treatment were measured using ImageJ (https://imagej.nih.gov/ij/, accessed date: 8 January 2022). The data were visualized using the GraphPad Prism v. 8 software (San Diego, CA, USA).

### 4.7. Spot Test Assay

To assess the susceptibility to different pH values in the case of the selected *Cryptococcus* sp. strains and to compare the relative susceptibility of several strains, the cells were grown to mid-log phase, diluted to OD_600_ ≈ 0.25 and spotted (3 μL) in 10-fold serial dilutions (10^0^, 10^−1^, 10^−2^ and 10^−3^) onto the YPD plates with different pH. Plates were incubated at 30 °C and photographed after 72 h or 96 h, depending when the phenotypic effect was most visible. The susceptibility assays were repeated a minimum of two times. Differences in growth show variability of the tested strains in their susceptibility to pH [29].

### 4.8. Statistical Analysis

To test differences between the median of total cell body (cell body with the capsule) or cell body, or the capsule (total cell body diameter minus cell body diameter and divided by 2) diameter, first the Normality Kolmogorov-Smirnov test was performed and subsequently, the one-way ANOVA (for normally distributed data) or Mann–Whitney and Kluskal–Wallis tests (non-parametric) were performed with the use of GraphPad Prism v. 8 software (San Diego, CA, USA).

## Figures and Tables

**Table 1:** Cell density, pH values and percent (%) of Titan cells after 48 h of incubation according to conditions ^#^ described by Dambuza et al. [18] depending on initial inoculum and tested strains of the *Cryptococcus neoformans*/*C. gattii* species complex.

Tested Strain	Cell Density *		Titan Cells (%)	pH **		Cell Density *		Titan Cells (%)	pH **	
	T_0_	T_48_		T_0_	T_48_	T_0_	T_48_		T_0_	T_48_
*Cryptococcus neoformans* H99	1.15 ± 0.2 × 10^3^	9.2 ± 1.3 × 10^5^	12%	7.2	7.05	1.13 ± 0.4 × 10^4^	3.2 ± 0.2 × 10^8^	7%	7.2	6.84
*Cryptococcus deuterogattii* R265	1.05 ± 0.3 × 10^3^	3.1 ± 0.5 × 10^5^	21%	7.2	7.08	1.04 ± 0.2 × 10^4^	1.6 ± 0.4 × 10^8^	13%	7.2	6.91
*Cryptococcus neoformans* MD31	1.1 ± 0.1 × 10^3^	5.9 ± 0.8 × 10^5^	18%	7.2	7.13	1.07 ± 0.3 × 10^4^	1.3 ± 0.3 × 10^7^	11%	7.2	7.04
*Cryptococcus bacillisporus* B4546	1.08 ± 0.1 × 10^3^	5.7 ± 0.3 × 10^4^	78%	7.2	7.18	1.17 ± 0.1 × 10^4^	8.2 ± 0.1 × 10^5^	75%	7.2	7.12

* determined based on colony counting (on YPD medium) after 48 h of incubation according to Dambuza et al. [18] protocol (results obtained from three independent repetitions); ** measured at the beginning of experiment (T_0_) and after 48 h of incubation (T_48_); **^#^** Conditions of incubation: 37 °C, 5% CO_2_, 48 h, in 10% FBS in PBS not buffered, according to protocol by Dambuza et al. [18].

**Table 2:** Strains used in this study, their source, place of the first isolation and the newest taxonomic position ^#^.

Strain	Source ^(1−3)^, Primary Isolation
Cryptococcus albidus ^a^, MD22.	^I^ Interdigital spaces of the feet, component of the skin mycobiome (healthy male, Poland)
^1^*Cryptococcus aspenensis*^b^, DS570	^II^*Populus tremuloides*, New York State (USA)
^2^*Cryptococcus aspenensis*^b^, DS569	^II^*Populus tremuloides*, New York State (USA)
*Cryptococcus curvatus*^c^, MD120/2009	^I^ Soil, Olsztyn, Poland
*C. deuterogattii*^d^ R265 (CBS10514)	^III^ Bronchial washings of infected patient from the Vancouver Island, British Columbia, Canada, VGIIa genotype
*Cryptococcus bacillisporus* WM161	^II^ Environmental isolate, San Diego CA, USA, VGIIIa genotype
*Cryptococcus bacillisporus* B4546	^II^ VGIIb genotype
*Cryptococcus bacillisporus* NIH312	^II^ Clinical isolate from cerebrospinal fluid, VGIIIb genotype
*Cryptococcus kuetzingii*^a,^* MUCL27749	^II^ Intestinal tract, *Blatta orientalis*
^1^*Cryptococcus laurentii*^e^, DS619	^II^*Picea abies*, New York State (USA)
^2^*Cryptococcus laurentii*^e^, DS288	^II^*Colophospermum mopane*, Botswana
^1^*Cryptococcus neoformans*^f^ H99	^II^ Male patient with Hodgkin’s disease, New York, USA
^2^*Cryptococcus neoformans*^f^ MD31	^I^ Bronchoalveolar lavage (BAL) of patient with AIDS, Poland
^1^*Cryptococcusterrestris*^g^, DS291	^II^*Colophospermum mopane*, Botswana
^2^*Cryptococcusterrestris*^g^, DS233	^II^*Colophospermum mopane*, Botswana
^3^*Cryptococcusterrestris*^g^, DS234	^II^*Colophospermum mopane*, Botswana
^4^*Cryptococcusterrestris*^g^, DS290	^II^*Colophospermum mopane*, Botswana
*Cryptococcus terreus*^h^, DUMC177.8	^II^ Soil, New Zealand
^1^*Cryptococcus uniguttulatus*^i^, MD29	^I^ Interdigital spaces of the feet, mycobiome (healthy female, Poland)
^2^*Cryptococcus uniguttulatus*^i^, MD26	^I^ Soil, Wroclaw, Poland

Source of strains: ^I^ L. Kozubowski collection, Clemson University, Clemson, SC, USA; ^II^ kindly provided by J. Heitman, Duke University, Durham, NC, USA; ^III^ gift from H. de Cock, Utrecht University, Utrecht, The Netherlands; ^1–4^ Roman numerals in superscript are compatible with those indicated in Figure 1A; ^#^ Internet source: http://www.indexfungorum.org/names/names.asp (accessed date: 20 June 2022). Actual taxonomic position: ^a^ *Naganishia albida*; ^b^ *Papiliotrema aspenensis*; ^c^ *Cutaneotrichosporon curvatum*; ^d^ *Cryptococcus gattii*; ^e^ *Papiliotrema laurentii*; ^f^ *Cryptococcus neoformans*; ^g^ *Papiliotrema terrestris*; ^h^ *Solicoccozyma terrea*; ^i^ *Filobasidium uniguttulatum*; * also known as *Cryptococcus albidus* var. *kuetzingii* (Fell & Phaff) Fonseca, Scorzetti & Fell.

## Data Availability

Not applicable.
